# Navigating intersectoral collaboration in nutrition programming: implementors’ perspectives from Assam, India

**DOI:** 10.1186/s13690-024-01312-6

**Published:** 2024-06-07

**Authors:** Praveenkumar Aivalli, Brynne Gilmore, Prashanth Nuggehalli Srinivas, Aoife De Brún

**Affiliations:** 1 UCD Centre for Interdisciplinary Research Education and Innovation in Health Systems (UCD IRIS Centre), School of Nursing Midwifery and Health Systems, University College Dublin, Dublin, Ireland; 2 School of Nursing Midwifery and Health Systems, University College Dublin, Dublin, Ireland; 3 Institute of Public Health Bangalore, Bangalore, India; 4 Guwahati, India

**Keywords:** Intersectoral collaboration, Nutrition, Policy implementation, Power dynamics

## Abstract

**Background:**

There is a growing interest in the use of intersectoral collaborative (ISC) approaches to address complex health-related issues. However, relatively little empirical research exists on the challenges of implementing, fostering and sustaining these approaches. Our study explores the perceptions and experiences of programme implementers regarding the implementation of an ISC approach, focusing on a case study of nutrition programming in Assam, India.

**Methods:**

We conducted qualitative semi-structured face-to-face in-depth interviews with eleven programme implementers from two selected districts of Assam, India. These participants were purposefully sampled to provide a comprehensive understanding of the experiences of implementing intersectoral collaboration. Following the interviews, an inductive thematic analysis was performed on the collected data.

**Results:**

The study identified three main themes: operationalisation of ISC in daily practice, facilitators of ISC, and barriers to effective ISC. These were further broken down into six subthemes: defined sectoral mandates, leadership dynamics, interpersonal relationships and engagement, collective vision and oversight, resource allocation, and power dynamics. These findings highlight the complexity of ISC, focusing on the important structural and relational aspects at the macro, meso, and micro levels. Interpersonal relationships and power dynamics among stakeholders substantially influenced ISC formation in both the districts.

**Conclusion:**

Despite challenges, there is ongoing interest in establishing ISC in nutrition programming, supported by political development agendas. Success relies on clarifying sectoral roles, addressing power dynamics, and engaging stakeholders systematically. Actionable plans with measurable targets are crucial for promoting and sustaining ISC, ensuring positive programme outcomes. The insights from our study provide valuable guidance for global health practitioners and policymakers dealing with similar challenges, emphasising the urgent need for comprehensive research given the lack of universally recognised policies in the realm of ISC in global health practice.

## Key messages

The primary message from this research is the recognition that the success of ISC in health system goes beyond mere structural arrangements and policy directives. It is profoundly influenced by the quality of interpersonal relationships, leadership styles, and the equitable distribution of power and resources. Acknowledging and nurturing these softer aspects can significantly enhance the effectiveness and sustainability of ISC efforts, leading to more successful health interventions and outcomes.

**Table:** 

**Textbox 1. Contributions to the literature**
• Operationalising intersectoral policies in developing countries remains poorly understood, impeding effective implementation efforts.
• The success of intersectoral collaboration within health systems relies on more than just structural arrangements and policy directives; it hinges crucially on the equitable distribution of power and resources. Recognising and nurturing these softer aspects is essential for enhancing the effectiveness and sustainability.
• By combining real-world experiences with theoretical frameworks, this study enhances our understanding of successful sectoral collaborations. It offers valuable insights that guide the development of future collaborative efforts and policies in diverse social and political contexts.

## Background

The term intersectoral collaboration (ISC) frequently refers to collective actions involving more than one specialised agency performing different roles for a common goal or purpose. Internationally, there have been growing calls for ISC [1, 2], and it is central to several of the UN’s sustainable development goals [3]. ISC is mentioned in Articles VII and VIII of the Alma Ata Declaration of 1978: “All governments should formulate national policies, strategies, and plans of action to launch and sustain primary health care as part of a comprehensive national health system and in coordination with other sectors [4]. The World Health Organisation (WHO) has also explicitly acknowledged the necessity of ISC in their statement on Intersectoral Action for Health, which calls for “a recognised relationship between part or parts of the health sector with parts of another sector which has been formed to take action on an issue to achieve health outcomes (or intermediate health outcomes) in a way that is more effective, efficient or sustainable than could be achieved by the health sector acting alone” [5]. These policy statements recognise that health programming implementation and success depend on multiple actors and sectors coordinating and collaborating to meet the shared goals of the programme. Statements including Primary Health Care (PHC), Millennium Development Goals (MDG) and Sustainable Development Goals (SDGs), also consider ISC as one of the most necessary principles for the health system [6–8]. The term ‘intersectoral collaboration’ has been used synonymously with concepts such as integration, collaboration, partnership, coordination and cooperation [9, 10]. For the purpose of this study, we define ISC as “strategic and coordinated policy decisions and programme actions in multiple sectors, such as health, agriculture, education, rural development, public health and women’s empowerment, to achieve a common goal of reduced undernutrition.”

There is a growing interest in the use of ISC to address complex and fundamental health needs, such as adequate nutrition [11–14]. National service delivery programmes, such as those to address issues like malnutrition, are immensely complex and require intersectoral action to tackle the scope of the challenge [15–19]. Malnutrition remains a significant problem in Low- and Middle-Income Countries and can increase the risk of morbidity, and mortality, especially among children [20, 21]. A review of the international literature over the past five decades indicates that the median case fatality from severe malnutrition has remained unchanged over this period and is typically 20–30% [22]. The field of nutrition recognises the importance of nutrition-specific actions, such as behavioural interventions to improve feeding, care, and hygiene practices; and interventions to deliver micronutrients, to improve maternal nutrition, and to prevent and treat illnesses and severe malnutrition [23]. The UNICEF 2015 conceptual framework of determinants of undernutrition identifies a set of immediate, underlying and basic causes of undernutrition [24]. While disease, household food insecurity, vulnerable living environment, as well as poor health care access and practices are identified as immediate causes of undernutrition; further, at the meso-level, these factors are influenced by a range of social, economic, and political factor and processes. The field also recognises the role of interventions and actions to address critical issues such as household poverty, food security, social equity, women’s empowerment, and other underlying factors. Ensuring that all key actions for nutrition are implemented in turn necessitates collaborative action between the fields of nutrition, health, agriculture, livelihoods, and women’s empowerment. The relationships between these various fields or sectors and their potential for improving nutrition have previously been well explained through multiple pathways [25]. These pathways explain the linkages between different sectors concerning creating enabling conditions for improved nutrition. Overall, the conceptual frameworks put forward that effective ISC is desirable and that it either exists (in ideal situations) or can be formed through a set of strategic mechanisms related to policies and programmes [26]. Several studies have reported that realising effective ISC across various sectors and delivering collaborative actions at the community level is perhaps one of the most significant challenges to improving child undernutrition [19, 25, 27–29].

### ISC and the nutrition programme in India

India is one country that has made an explicit commitment to integrate ISC into its development agenda, particularly on nutrition, health and well-being, through designing and launching a new National Nutrition Mission in 2018 [30]. The mission aims to reduce malnutrition through a lifecycle concept, adopting a synergised and result-oriented approach. Implemented by the Ministry of Women and Child Development [31], Government of India, the mission’s target is to reduce stunting in children 0–6 years of age from 38.4% to 25% by 2022. It also aims to reduce anaemia among women and adolescent girls aged 15–49 years and reduce low birth weight. To ensure smooth implementation and operation in every state, a State Programme Management Unit at the state level and a District Programme Management Unit at district level have been established with additional human resources to support implementation [32]. The NNM integrates ISC as a key component for implementing nutritional interventions across India. This approach is outlined in the ‘Intersectoral Nutrition Action Plans’ at the state, district, block, and village levels. It involves coordination among various sectors such as Health and Family Welfare, Water and Sanitation, and Rural Development. At the national level, collaboration is overseen by the National Council for Nutrition and the Executive Committee for NNM, comprising members from all relevant sectors. At the state level, collaboration is facilitated through state, district, and block level committees led by the top administrative officials. At the village level, the Village Health Sanitation and Nutrition Day serves as the platform for collaboration, focusing on service delivery by health and women and child development sectors. This hierarchical and inter-sectoral approach aims to enhance the efficacy of nutrition interventions across India. Operationalising ISC under the new NNM begins with identifying existing nutrition-specific and sensitive schemes being implemented by different sectors at the state, district, and block levels. Nutrition-specific interventions refer to interventions that address the immediate determinants of foetal and child nutrition and development. These include micronutrient supplementation, exclusive breastfeeding, dietary diversity promotion, treatment of severely acute malnourished children, disease prevention and management, nutrition emergencies, and food supply and fortification [31]. Nutrition-sensitive interventions influence the underlying determinants of nutrition. These include water sanitation and hygiene, immunisation, child protection, schooling, early child development, maternal mental health, food security, safe and hygienic environment, family planning services, social safety nets, and women’s empowerment [32]. The collaboration committees are expected to develop annual collaboration action plans considering key nutrition actions to be carried out by the different sectors. The committee reviews and monitors progress against these indicators quarterly, identifies gaps, and introduces effective interventions or innovations to address these gaps. Despite strong calls globally and perceived benefits of ISC, establishing intersectoral collaborative mechanisms has been problematic. The challenges to ISC are thought to be more acute in LMICs where institutions are frequently weak and fragmented, even within the health sector, which can undermine coordination [28]. The potential merits of ISC, therefore, still need to be discovered in the absence of empirical assessments of the prospective roles of relevant sectors for reinforced action and shared accountability [33, 34]. Evidence also suggests that the challenges to building intersectoral commitment and action are substantial, as are the challenges to operationalising intersectoral actions for nutrition [10, 35]. To date, studies on the development of collaborations in healthcare have emphasised the conditions that give rise to their formation [36, 37], and the few available studies have highlighted the facilitators and barriers in implementing ISC [26, 38–41]. Little empirical research has examined how to foster and sustain collaboration during or after stages of development [36].

### Gaps in WHO definition of ISC

Additionally, the WHO’s definition of ISC primarily focuses on the collaboration between the health sector and other sectors, such as education, environment, and agriculture [42]. While this is important, there is a need to expand the definition to include collaboration between different sectors within the health system itself. Moreover, WHO’s existing guidance on ISC operationalisation remains ambiguous, hindering the translation of policy into effective practice. The complexities of operationalising ISC within intersectoral health interventions like the NNM call for nuanced exploration beyond formal policies.

#### Governance mechanisms in intersectoral health approaches: a theoretical perspective

Effective governance mechanisms are essential for navigating the complex interplay of administrative structures, political dynamics, and diverse stakeholder engagements in intersectoral health initiatives. This section explores the foundational framework of governance mechanisms in ISC, highlighting their role in promoting coordination, cooperation, and accountability among stakeholders involved in health programming. Theoretical frameworks such as network governance theory [43] and collaborative governance theory [44] provide valuable insights into the multifaceted nature of governance mechanisms, highlighting the importance of fostering partnerships, building trust, and facilitating adaptive governance structures to address dynamic health challenges. At the administrative level, tools such as interagency coordination committees, task forces, and joint planning mechanisms facilitate intersectoral dialogue and decision-making [45]. These administrative tools provide platforms for stakeholders to align goals, share resources, and collectively address health priorities. Moreover, political engagement is integral to governance mechanisms in ISC, with theories of political economy [46] emphasising the influence of power dynamics, interests, and incentives on intersectoral policy processes. Effective political engagement entails leveraging political will, navigating competing interests, and advocating for policy coherence to sustain collaborative efforts over time. Additionally, governance mechanisms in ISC must encompass diverse stakeholders, including communities and civil society organisations [47]. Participatory governance approaches [48] stress the importance of engaging communities as active partners in decision-making processes, ensuring responsiveness to local needs and priorities. By integrating theoretical insights on administrative tools, political engagement, and stakeholder diversity, this section offers a comprehensive understanding of governance mechanisms in ISC, informing strategies for fostering effective intersectoral health approaches. In the context of the nutrition mission in Assam, these governance mechanisms are critical for promoting ISC to address malnutrition and improve public health outcomes.

This qualitative study delves into the lived realities of ISC operationalisation in Assam, India. Through in-depth interviews with programme managers and implementers, we aim to uncover the factors that enable or hinder the formation, implementation, and sustainability of ISC within the nutrition programme. By bridging the gap between theoretical frameworks and real-world experiences, this study seeks to enrich our understanding of ISC beyond policy pronouncements. The insights gained have the potential to inform and enhance the development and implementation of more effective intersectoral health interventions, not only in nutrition programming but also in similar intersectoral programmes globally.

### Aims and objective

The aim of the study is to gain insight into the perspectives and experiences of programme planners and implementors involved in implementing nutrition interventions on their understanding of the ISC approach and operationalisation in day-to-day work, including factors that facilitate and hinder its implementation.

## Methods

### Study setting

Two geopolitically and culturally diverse districts of Assam, India were purposefully selected. The selection of two districts was based geographic diversity, population demographics, and accessibility. These districts were chosen to provide a representative sample while ensuring feasibility and resource efficiency. Each district is further divided into sub-districts for administrative purposes. District A is slightly smaller compared to District B in terms of geographical area and population. With respect to nutrition indicators, District B is slightly better performing compared to District A. The demographic and nutritional characteristics of these districts are outlined in Table 1. These two districts offer valuable insights into intersectoral collaboration dynamics within Assam’s context, which can inform similar initiatives in comparable settings.

**Table 1:** Demographic and nutritional characteristics of districts A and B based on NFHS-5 Data (2019–2020)

**Characteristics**	**District A**	**District B**
*Demographic details (Approx. values)*		
Geographical area	2,000 Sq.Km	3,000 Sq.Km
Population	0.8 to 1 million	1 to 1.5 million
Literacy rate	65 to 70%	70 to 80%
*Nutrition indicators as per National Family Health Survey five 2019–2020 (Approx. values)*		
Stunting	30%	25%
Wasting	20%	20%
Underweight	30%	32%
Anaemia among under-five children	80%	70%

#### Context of the research

Conceptualising and implementing ISC in India poses unique challenges and opportunities that differ from those in other parts of the world. India’s complex sociopolitical landscape, with its diverse cultural norms, bureaucratic structures, and resource limitations, influences how collaborative efforts unfold. Unlike many Western contexts, where ISC typically functions within clear institutional frameworks and established governance structures, India’s decentralised governance and varied administrative setups require innovative collaboration strategies across sectors [49]. Moreover, India’s pluralistic society, with its rich tapestry of languages, religions, and traditions, requires nuanced strategies for fostering inclusive participation and addressing disparate stakeholder interests [50]. Additionally, the scale and scope of India’s developmental challenges, including poverty, inequality, and healthcare disparities, underscore the imperative for effective ISC to address multifaceted issues spanning health, education, social welfare, and economic development [51]. Furthermore, India’s historical legacy of colonialism and subsequent efforts towards nation-building have left indelible imprints on governance structures and policy frameworks, influencing the conceptualisation and implementation of collaborative initiatives at the grassroots level [52]. Therefore, a nuanced understanding of India’s context is crucial for effectively conceptualising and implementing ISC initiatives. Tailoring strategies to local realities is necessary to harness the potential for transformative change in addressing complex societal challenges.

### Participants

A cohort of eleven experienced programme implementers, responsible for executing intersectoral nutrition interventions in two regions of Assam, was carefully selected for this study. While the total complement of programme managers operating within these areas comprises eleven individuals, a deliberate decision was made to include all eleven programme implementers in the study cohort. Table 2 outlines demographic and profiles of district and block programme implementors in both districts. Determination of the sample size was rigorously informed by the foundational principle of information power [53], a concept deeply entrenched within qualitative research paradigms, which accentuates the qualitative richness and contextual relevance of data garnered from participants. Additionally, the deliberate selection of implementers from both district and block levels ensured a comprehensive understanding from diverse operational viewpoints. Participants were chosen for their extensive programme management experience, providing valuable insights into the operational nuances of intersectoral coordination, particularly relevant given the mission’s recent emphasis on such approaches. In qualitative research paradigms, depth and nuance of data often hold greater weight than sample size. A smaller, experienced cohort can yield significant insights into complex processes. Employing a purposive sampling strategy, participants were judiciously selected based on their extensive professional experience in operationalising ISC within nutrition programming, ensuring a comprehensive representation of perspectives salient to the research objectives. Identified through purposive sampling, these eleven programme implementers were invited to participate in interviews with the lead researcher. Their narratives proved instrumental in delineating both the facilitators and barriers to effective ISC, ultimately providing a nuanced understanding critical for enhancing multi-sectoral public health strategies.

**Table 2:** Demographic and profiles of district and block programme implementors in districts A and B (2022–23)

	Age	Gender	Background	Years of experience
**District A**				
District facilitator	29	M	Masters in computer tech	7
Block 1	26	F	Bachelors In art and humanitarian	4
Block 2	27	M	Masters in computer tech	3
Block 3	29	F	Masters in computer tech	6
Block 4	28	M	Bachelors in social works	6
**District B**				
District facilitator	36	M	Masters in sociology	9
Block 1	31	F	Bachelors in home science	7
Block 2	34	M	Bachelors in business administration	8
Block 3	29	F	Bachelors in arts and humanitarian	7
Block 4	28	M	Bachelors in social work	5
Block 5	38	M	Master in humanitarian works	8

### Data collection and analysis

Semi-structured, in-depth interviews were conducted face-to-face with participants who had willingly agreed to take part in the study from February 2022 to April 2022. All interviews were carried out by the first author, an experienced qualitative researcher with a background in nutrition programme implementation. Interviews were conducted in Hindi and Assamese languages. Interviews were audio recorded with the informed consent of participants. All interviews were transcribed, translated to English, and manually coded according to the emerging themes and topics, from which key narratives and storylines were developed following Braun and Clarke’s guide to thematic analysis [54]. Consistent with best practice, the first author read the interview transcripts repeatedly to ensure familiarisation and immersion in the data. This was followed by a line-by-line coding of each transcript. The generated codes were organised into themes, which were revised iteratively, taking due cognisance of internal heterogeneity and external heterogeneity. Through this iterative process, relevant themes identified, discussed, refined, agreed upon, and finalised with the wider research team.

## Results

### Section A: participant demographics and sectoral mapping

This study drew on the insights of eleven programme implementers from diverse backgrounds within Assam’s NNM. Their expertise, spanning district and block levels, covered various programmes and management functions, including planning, execution, and monitoring. This carefully selected group provided a comprehensive understanding of ISC within the mission from multiple operational perspectives. Participants’ firsthand accounts illuminated the practicalities of implementing such collaboration, offering valuable lessons for future programme design. Additionally, their varied backgrounds and expertise added significant depth and credibility to the study’s findings, illustrating the complexities of executing a large-scale, intersectoral public health programme in Assam.

**Fig. 1:**
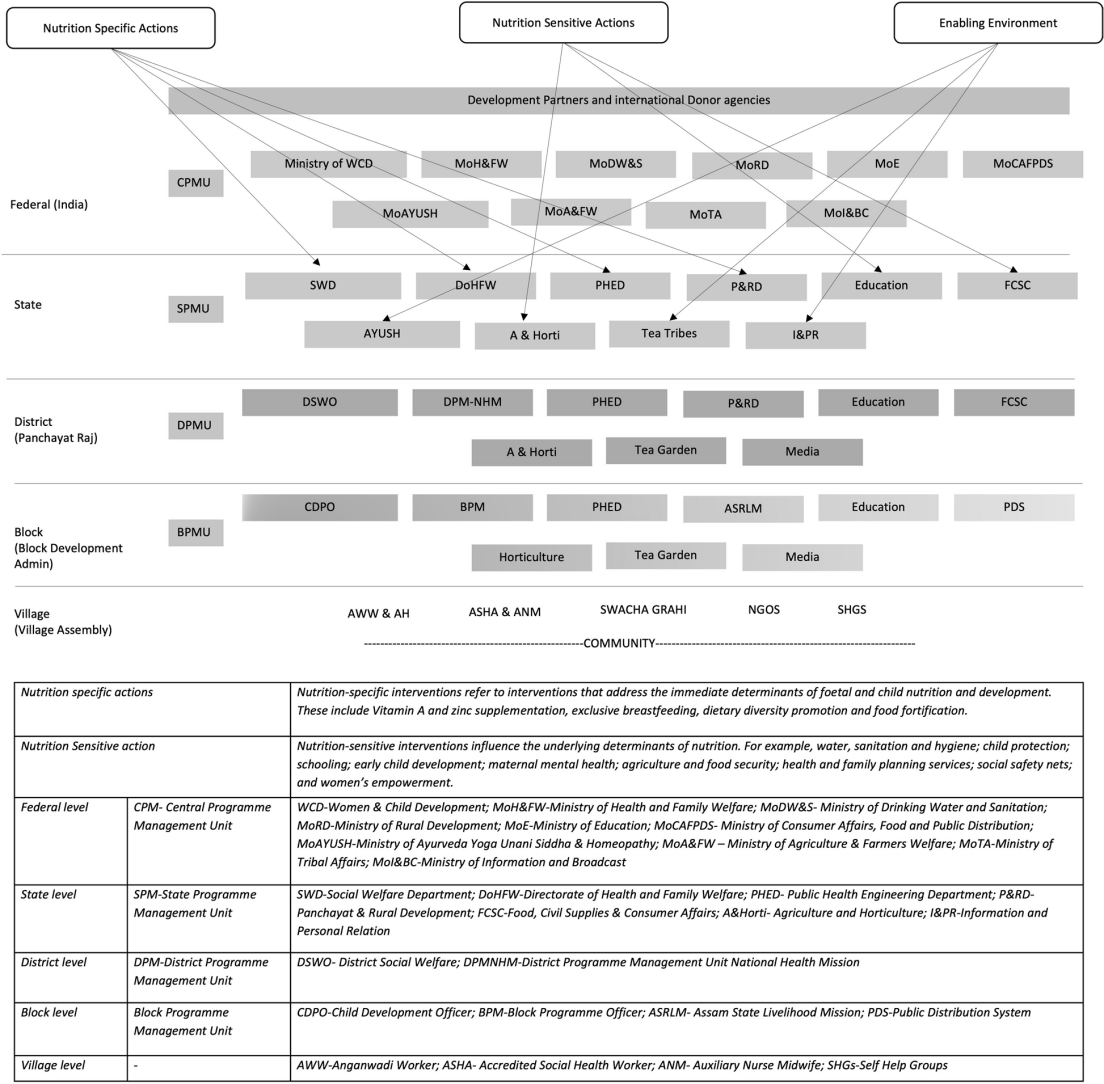
Different sectors involved in the implementation of intersectoral nutrition interventions at different administrative levels

For the purpose of this study, a detailed mapping exercise was conducted with the participants to comprehensively understand the involvement of various sectors in the nutrition programme. This exercise was crucial in visualising the intricate network of sectoral collaboration required for the effective implementation of the mission within Assam. Participants, who were programme implementors from district and block levels, provided insights into the range of sectors they coordinate with on a day-to-day basis. These insights were supplemented by an extensive review of relevant guidelines and policies associated with the NNM. This approach ensured a thorough understanding of both the theoretical framework of the mission and its practical execution on the ground. The findings from this exercise were collated into a visual diagram (Fig. 1), which illustrates different sectors involved in the implementation of intersectoral nutrition interventions at different administrative levels. This representation serves to highlight the complexity and interconnectivity of the sectors, showcasing how sectors such as health, education, agriculture, women’s empowerment, public health, private sector, non-governmental organisations academic institutions and rural development come together in a concerted effort to address nutritional challenges. The mapping clearly demonstrated that the NNM is not just the purview of health or nutrition sectors but is a multifaceted initiative requiring coordinated efforts across various sectors. Each sector contributes unique expertise and resources, playing a critical role in the holistic approach needed to tackle the multifactorial issue of malnutrition. The exercise highlighted the need for effective intersectoral communication and collaboration. It emphasised the importance of each sector understanding its role and how it fits into the bigger picture of the mission. This mapping serves as a valuable tool for programme implementers and stakeholders, providing a clear visual guide to the complex network of collaborations essential for the success of the NNM.

### Section B: thematic analysis of the findings

Thematic analysis of the interviews revealed three key themes and six subthemes related to ISC:

- **Theme 1: Operationalisation of ISC in daily practice**
- **Theme 2: Facilitators**
  - ***Sub themes:**** Defined sectoral mandates, Leadership dynamics, Interpersonal relation and engagement*
- **Theme 3: Barriers**
  - ***Sub themes:**** Lack of collective vision and oversight, unfair resource allocation, and power dynamics*

### Theme 1: operationalisation of ISC in daily practice

First theme delves into the practical enactment of within the routine functioning of health programmes, particularly nutritional interventions. This theme is crucial as it bridges the gap between theoretical constructs of ISC and their tangible execution.

Participants consistently stressed the indispensability of effective ISC for the success of nutrition programmes. They illuminated the notion that programmes such as Community Management of Acute Malnourished Children (CMAM), Water and Sanitation Hygiene [55], and Weekly Iron and Folic acid Supplementation (WIFS) require a synergistic effort across various sectors to achieve their objectives. This aligns with the collaborative governance framework. For instance, participant 4 described how their understanding of the need for collaboration across sectors grew, showing how collaborative governance principles work in practice, a participant recounted:

*“Nutrition interventions like CMAM (Community Management of Acute Malnourished Children), WASH (Water, Sanitation And Hygiene), and IFA (Iron and Folic Acid) consumption in schools require sectors to work in close coordination; otherwise, these programmes are not successful. When I joined first, I thought it was a data entry and compiling type of job, but I later learnt that there are several sectors involved to make nutrition intervention successful in the field.” (Participant 4)*

On the other hand, a few participants reported that despite an agreement on the importance of collaboration, the operationalisation of these agreements into tangible outcomes often encounters stumbling blocks. One of the participant pointed out the challenges in navigating the differing work cultures and priorities of each sector. The sentiment expressed here is that while reaching a consensus on objectives is feasible in the structured environment of a meeting, the diverse expertise and institutional cultures present challenges in the translation of these strategies into effective programme delivery. Reflecting on their experiences, one participant recalled

*“Establishing intersectoral collaboration is challenging due to different work cultures between sectors. All sectors have their own priorities. Every sector is expert in their own subject, bringing all their expertise to the table and agreeing for achieving common goals is easy in meetings, but it’s difficult while operationalising it because at every level different actors are involved.” (Participant 3)*

Additionally, a participant emphasised the reliance of ISC on not just coordination, but timely action. Participants also stressed the need for a supportive network and highlighted how interpersonal relationships can accelerate or hinder progress. The quote from participant 5, suggests that when good relationships exist, work is expedited, implying that social capital is a significant currency in ISC.

*“We need support in most of these activities, the entire mission itself is based on coordination, but sometimes it is difficult to get the work done timely manner. Because other sectors will have other meetings and work, our work is not important to them. They say yes to our faces, but the process gets delayed unnecessarily; if we have a good connection with these officials, work will be done immediately.” (Participant 5)*

Another participant reported on a critical issue affecting the operationalisation of intersectoral nutrition interventions in schools, particularly the administration of IFA supplements, which are essential for addressing nutritional deficiencies in children. The participant’s account reveals a troubling shortage of IFA stocks in schools, which directly obstructs the programme’s execution. This shortage is compounded by a hesitancy among teachers to distribute the tablets to children—a task for which they attend training and meetings but hesitate to perform due to fears of potential health risks to the students, particularly adolescent girls. The participant also sheds light on the resistance from parents, who express their mistrust in free supplied medications by complaining to teachers and advising against the use of these supplements for their children. A participant elaborated

*“There were no stock of IFAs in school that I visited last month. Teachers are not bothered to administer the IFA tablets it to children, they will attend all our meetings, but when it comes to administering the tables, they are hesitant and scared that if anything would happen to adolescent girls. There also have been instances of parents complaining to teachers not to give free medicines to their children, and they see a good doctor instead.” (Participant 7)*

### Theme 2: facilitators of ISC

Theme 2 explores the key elements that support and improve ISC within nutrition programmes. This theme delves into implementers’ perspectives on the dynamics and conditions necessary for effective ISC. It focuses on three subthemes: defining sectoral mandates, which involves clarifying roles and responsibilities among diverse stakeholders; leadership dynamics, which looks at how different leadership styles and strategies impact ISC effectiveness; and interpersonal relations and engagement, which highlights the importance of building and maintaining strong, collaborative relationships across sectors.

#### Defined sectoral mandates

Several participants stressed the importance of well-defined sectoral mandates in the operationalisation of ISC. A participant emphasised the need for a systematic approach, where sectors are not only mapped out with clearly delineated roles but also are held accountable through regular follow-ups and meetings. This participant elaborated that such a systematic approach is not just about assigning tasks; it is about cultivating a sense of ownership and responsibility towards shared goals. As an example, one participant described

*“Firstly, there should be a mapping of sectors and allocating roles for each sector with achievable clear targets. And there should be strict follow up of these gaols in monthly meetings then only these sectors own these gaols, for example we have CMAM programme which states which sector looks after what task it is easier to operationalise such interventions.” (Participant 8)*

The mention of the CMAM programme as an example highlights how clear, achievable targets assigned to specific sectors can streamline the process, making the operationalisation of such interventions more feasible and efficient according to the participant. The participant also points out that the effectiveness of such a system is enhanced when there are regular check-ins on progress, ideally through monthly meetings. These meetings serve as a platform for different sectors to update each other on their progress towards meeting their targets, discuss challenges, and coordinate efforts.

#### Interpersonal relationships

The importance of interpersonal relationships in fostering successful ISC is highlighted by many participants. Participant 11 particularly emphasises the crucial role of personal connections in ISC engagements and the significance of consistent communication across sectors. Furthermore, the participant highlighted the need to pay attention to others’ priorities, reflecting individuals’ desire for positive social identity through alignment with group norms and goals. By actively listening to and supporting the initiatives of other sectors, participant 11 highlights the establishment of reciprocity, where mutual benefit promotes ongoing collaboration. This ‘give-and-take’ policy represents a fundamental principle of effective ISC. From this perspective, interpersonal relationships and communication emerge as key mechanisms that facilitate ISC by fostering trust, resource exchange, goal alignment, and reciprocal cooperation.

*“Personal relations matter a lot; if you are good, they are good. In my case I meet them constantly and update them about our progress also, sometimes I have to listen to their priorities. sometimes I end up extending my support to their sectoral programmes. It’s a kind of give-and-take policy. Effective collaboration also depends a lot on officers’ attitude and trust.” (Participant 11)*

The participant also touches on the significance of attitude and trust among officers. They suggest that the disposition of the individuals involved can greatly influence the collaborative process. A positive attitude and the establishment of trust are pinpointed as key factors that can either facilitate or hinder the effectiveness of working together across different sectors.

Furthering this understanding, participant 2, underscores the vital importance of one’s approach and the cultivation of interpersonal relationships for successful intersectoral collaboration. Participant stress the impact of their own continuous and deliberate efforts to connect with other sector heads through various forms of communication. By engaging in repeated interactions and holding regular meetings, whether it be over the phone or during informal tea sessions, the participant has been able to foster stronger connections and a sense of camaraderie with their counterparts. This participant attributes the improvement in their working rapport within the block to these sustained efforts at relationship building. They highlight that these are not occasional or one-off meetings but a consistent and focused strategy to enhance mutual understanding and cooperation among the sectors.

*“A lot depends on our approach and interpersonal relationships; I had repeated interactions and regular meetings with different sectors heads over the phone and over a tea. Repeated meetings and familiarisation have improved my rapport in the block. Our District Commissioner sir also very much promoted teamwork. He treats all the sector equally.” (Participant 2)*

Additionally, the participant notes the positive influence of leadership in promoting a collaborative environment. They praise the District Commissioner for his role in advocating for teamwork and for treating every sector equally. According to the participant, the District Commissioner’s actions have significantly contributed to creating an atmosphere where teamwork is not only encouraged but is also a lived value.

#### Leadership dynamics

Effective leadership played a crucial role in guiding collective action towards shared health goals. Proactive leadership, drawing from transformational principles, fostered inclusivity and unity among stakeholders. Participant 10 highlighted this through their Block Development Officer (BDO), who organised inclusive intersectoral meetings using tools like monthly meetings and committees. The BDO ensured participation from all sector heads, promoting shared ownership and accountability. Sharing meeting minutes with those absent fostered transparency and kept everyone informed. By actively encouraging participation and resolving conflicts, the BDO promoted open communication and problem-solving. The BDO’s repeated interactions with sectors like education emphasised the importance of building relationships and trust to overcome initial barriers to collaboration. These strategies, reflecting shared and distributive leadership principles, collectively enhanced engagement, communication, and cooperation among stakeholders in ISC initiatives, aligning efforts towards common health outcomes, as supported by social identity principles.

*“Our BDO sir ensures all sectorial heads participate in the meeting, even if some sector did not attend due to some other engagement, he makes sure that the minutes reach them. Sir give chance to everyone to speak and resolve any conflicts if arise during meeting. For example, in case of WIFS programme implementation in school, the education sectors presence was minimal thinking that the WIFS programme is health sectors programme, but after our BDO sir repeated interaction with them now a days they are attending regularly and participate in discussions.” (Participant 10)*

The participant also highlights the BDO’s commitment to democratic principles during meetings, where every attendee is given the opportunity to speak and any conflicts that arise are addressed through dialogue. This approach has been particularly effective in the case of the WIFS programme in schools, where initially, the education sector’s involvement was limited. The participant attributes the change in the education sector’s engagement to the BDO’s persistent efforts in encouraging their regular attendance and active participation in discussions. According to the participant, these efforts by the BDO have led to a significant improvement in the education sector’s collaboration in the WIFS programme, indicating a shift in their perception of the programme being solely a concern of the health sector.

### Theme 3: barriers for ISC

Three key barriers to effective ISC have emerged, each posing significant challenges to intersectoral initiatives. Firstly, having a collective vision and proper oversight is crucial for a unified approach and strong monitoring to ensure all sectors are working towards common goals. Secondly, fair resource allocation is essential for equitable distribution of resources, ensuring that all sectors are fully supported and engaged in ISC efforts. Lastly, power dynamics and hierarchical disparities create obstacles due to unequal power relations and structures, making it difficult to build trust and equitable partnerships across different organisational levels. This issue of power asymmetry highlights the disproportionate influence and control some sectors have over decision-making, hindering collaborative efforts.

#### Lack of shared vision

The participant 1 details specific operational issues encountered with collaboration committees, which are mandated by the ministry to facilitate intersectoral collaboration. They point out that, in practice, the regularity of these committee meetings falls short of the ministry’s directives. Instead of meeting consistently as intended, the committees convene primarily during special events or large-scale campaigns, which deviates from their original purpose of ensuring ongoing, systematic collaboration.

*“We have directives from the ministry for forming collaboration committees, these committees should meet regularly, but these committees meet if there are any special events like celebration or any mass movement campaign etc. There is no clarity on the nutrition target from the other sector except the health sector.” (Participant 1)*

Furthermore, the participant also expresses concern over the unclear targets related to nutrition outside the health sector. They note that while the health sector has delineated nutrition targets, there is a lack of clarity and communication regarding these goals from other sectors involved. According to the participants such absence of shared clarity on objectives undermines the potential for effective collaboration, as all sectors are not aligned in their understanding and pursuit of common targets in nutrition.

#### Unfair resource allocation

In addition to the lack of collective vision several participants raised the concern of equitable resource distribution which has been identified as a pivotal factor influencing the efficacy and sustainability of collaborative efforts. Participant 6, particularly noting the lack of financial support for collaboration meetings and training activities. This participant pointed out that while the health sector benefits from a robust funding stream, other sectors struggle with insufficient financial resources, hampering their ability to contribute to and participate in ISC activities. The participant elaborated,

*“No, we don’t get much funding for convergent meetings and training activities, but the health sector has a lot of funding. If they club some of their meeting and capacity-building session jointly with us, that would be beneficial. If this change happens at higher level and gets a joint letter for such collaborative events, it is easier to push the collaboration further down at the field level.” (Participant 6)*

From the participants perspective a joint letter or directive from higher authorities endorsing shared resource use for ISC-related activities could serve as a catalyst for this change. This would enable a more equitable distribution of resources, ensuring that all sectors can engage meaningly in ISC and that the collective impact on health programmes is not compromised by financial constraints.

#### Power dynamics

Power dynamics emerged as a recurring theme in discussions on ISC. Participants identified power dynamics as a major barrier to fair collaboration. They highlighted how the health sector often wields disproportionate power in collaborative efforts. This observation aligns with the concept of power asymmetry, where some actors or sectors have more control over decision-making and resource allocation in collaborative settings. One participant expressed.

*“I feel that the health sector is the most powerful compared to all other sectors. Because it has so many professional doctors, nurses and ANMs who work to cure the disease and save lives, our work in in front of them is insignificant. In some instances, the final call on referring malnourished children to NRC was taken only by health sector, because they are more qualified than us. Joint training with the health sector would boost our confidence in identifying malnourished children more accurately. This would also help the health sector trust our data.” (Participant 9)*

The participant expresses a perspective that the health sector holds a position of greater power and influence within the context of intersectoral collaboration, primarily due to the critical nature of its work and the professional qualifications of its personnel. They highlight that the sector employs numerous professionals such as doctors, nurses, and Auxiliary Nurse Midwives (ANMs), whose roles are directly associated with life-saving interventions and treating diseases. Participants also expressed that the prominence of the health sector overshadows the work done by professionals from other sectors to the point where their contributions can seem less significant. They note that in certain situations, such as when decisions are made about referring malnourished children to Nutrition Rehabilitation Centers (NRCs), it is the health sector that predominantly makes these decisions, given their higher qualifications and expertise in health matters. Additionally, participants also reported that joint training programmes could also help in building trust between the health sector and other sectors, by demonstrating the reliability and validity of the data provided by non-health sectors.

## Discussion

Our study explores the complex dynamics of ISC within the nutrition programme in Assam, focusing on the lived experiences and perspectives of programme implementers. This investigation is crucial for understanding how ISC operates, especially in translating policy into effective, on-the-ground actions. Our findings highlight the intricate nature of ISC, showcasing its structural and relational complexities at macro, meso, and micro levels. This aligns with network governance theory, which explains governance structures as networks of actors working together to achieve common goals [43]. Understanding ISC necessitates recognising the interplay of administrative structures, political dynamics, and stakeholder engagements, echoing the collaborative governance theory’s emphasis on inclusive decision-making and problem-solving [44]. A pivotal theme that emerged from our data is the significance of defined sectoral mandates in facilitating ISC, resonating with existing literature on policy frameworks and guidelines guiding intersectoral collaboration initiatives [56]. Governance mechanisms, including policy frameworks and institutional arrangements, play a pivotal role in establishing and enforcing sectoral mandates to ensure accountability and coherence in ISC efforts. Administrative structures, such as inter-ministerial committees, serve as platforms for high-level coordination and decision-making, illustrating the application of network governance principles in ISC [43]. Furthermore, our study identifies interpersonal relationships and engagement among stakeholders as crucial facilitators of ISC, echoing participatory governance approaches that emphasise active involvement and collaboration among diverse stakeholders [57]. Building trust, effective communication, and a shared understanding are essential for overcoming barriers and fostering collaboration. Civil society organisations and community representatives are vital for engaging the community and responding to local needs, aligning with the principles of participatory governance [57]. However, barriers such as power asymmetry, resource allocation challenges, and the lack of collective vision and oversight can hinder effective ISC, highlighting the need to address these issues systematically. Our study offers valuable insights into ISC in nutrition programming, revealing the complexities and challenges involved. By using theoretical frameworks such as network governance theory, collaborative governance theory, political economy, and participatory governance approaches, we provide a comprehensive understanding of the governance mechanisms and decision-making processes behind ISC initiatives. These insights are crucial for informing policy development and guiding future ISC efforts in global health practice.

Additionally, the findings of this study highlight the existence of a mandate for ISC within the NNM in the form of policy and guidelines, but in practice, however, there is limited joint planning and coordination due to sectoral priorities and competing interests by other sectors at all levels. Although most participants acknowledged and emphasised the value of intersectoral collaboration in planning and implementing nutrition interventions at different levels, due to a lack of supporting policies and institutional structure, it was challenging to achieve engagement and sustain the partnership across the sectors. Our results also indicate that the nature and extent of intersectoral collaboration varied at different system levels. While the district-level ISC efforts were found to be performing successfully to some extent, intersectoral coordination at the block level was rarely institutionalised, where most of the interventions go through final steps before it gets disseminated in the community. This finding concurred with results from another field study that highlighted the need for shared priority and regular action at all levels [9, 58]. Our study also revealed several important facilitators and barriers to successful collaboration across sectors. While consistent with the literature [9, 10, 27, 38, 59–68], our study identifies some facilitators and barriers are more important than others which the programmes like NNM need to consider and prioritise in operationalising in the field. For example, unclear sectoral roles, sectoral priorities, mistrust between sectors, poor monitoring and hierarchical institutional structures not only created barriers to ISC but led to rivalry and destroyed the spirit of collaboration for intersectoral action. Underlying socio-political, institutional and cultural context determines and shapes outcomes of intersectoral collaborative arrangement [62]. Hence, without sufficient consideration of important contextual factors, a wholesale or ‘one-size-fits-all’ implementation of ISC approaches could lead to failures rather than success. Our findings, such as shared vision, resource sharing, and personal relationships that have facilitated the ISC, are consistent with studies highlighting leadership and political commitment for better intersectoral action [9, 27, 60, 69]. Effective ISC requires a deliberate process with the alignment of a range of factors, including favourable initial starting conditions of partnerships, leadership, consensus, governance, and capacity, amongst others [28, 44, 70, 71]. Our study highlights the significant influence and dominance of the health sector in shaping policies. This aligns with research from Portugal, which shows that health decision-making processes are centralised and primarily focused within the health sector’s boundaries [72]. Reframing health objectives using non-health sector terminology is crucial to foster interdisciplinary collaboration and align public health priorities across governmental domains [73]. Additionally, within the ISC, continued support from activists, expert institutions, and civil society organisations is essential to drive social change processes, bridging the gap between policy formulation and population-level impact to promote a healthier society [74]. Lack of consensus regarding the roles of various sectors impacts ownership of the intervention, creating uncertainty regarding which governance or funding structures should be established, and this could result in a lack of coordinated action in programmes like NNM. Additionally, insufficient guidance on WHO definition for ISC significantly hinders its conceptualisation and operationalisation in country like India with its complex socio-political and healthcare system. India’s diverse governance, service delivery systems, and resource constraints further complicate ISC operationalisation, necessitating a clear framework for effective coordination, resource allocation, and monitoring. Without it, effectively assessing and enhancing ISC initiatives in India’s unique context remains a challenge.

### Dominance and power asymmetries in ISC

The findings of this study suggest that dominance and power asymmetries between the sectors emerged as significant barriers to intersectoral collaboration. In our analysis, we found that district-level and block-level intersectoral committee meetings are predominantly driven by the health sector reviewing numerical progress of indicators rather than comprehensive feedback or review of programmatic operations. Participants also reported that the health sector gets more advantages in terms of resource allocation, funding and skilled human resource when compared to other sectors. The trust issues between the sectors were also a significant barrier to ISC; this finding was consistent with a study highlighting barriers in operationalising one health approach in India [62]. Mistrust between the sectors impacts the spirit of teamwork and collaborative action. The domination of the health sector was perceptible in our results.

As per the guideline, usually, the sector’s head (from each sector) is the focal point person, who will be a senior bureaucrat from that sector who is less accessible than the mid-level operational personnel. Participants reported that approaching senior bureaucrats would sometimes become difficult due to their workload; however, most of the mid-level managers are accessible, but they do not have the authority to take a decision or commit on the sector’s behalf, which makes ISC a long and lengthy policy exercise. This can be addressed with a political commitment, strong leadership, clear delineation of sectoral goals, task shifting to mid-level managers, an explicit division of labour, and integrated accountability wherein the contributions of different sectors are considered to a greater extent. Review by external individuals who are not directly involved in policy or implementation can identify barriers such as vested interest and shared goals and lessons. Creating spaces to build familiarity and trust between the stakeholders, e.g., dedicating a fixed day in a month for intersectoral meeting, structuring these meetings, ensuring that each of the sector’s work is given adequate recognition and prominence, creating supportive mechanisms through appointing expert point persons to deal with collaboration related issues within respective sectors, tweaking goals to align the work structure of sectors are critical for implementors to work together and sustain the partnerships. Existing health policy should promote coordination across different sectors through joint committees, shared work plans, and pooled budgets which are crucial to intersectoral collaboration, as demonstrated in the health in all policy approaches [75]. The success of these initiatives depends on acknowledging and accommodating diverse and sometimes competing interests of other sectors. Intersectoral initiatives demand the creation of organisational cultures and ideologies that reward intersectoral efforts, providing incentives, and building informal networks across the sectors to foster shared values and trust.

### Limitations of the study

Several constraints are pertinent to our study. Initially, it is imperative to acknowledge that the research was confined to two purposively chosen districts, thus limiting the generalisability of the findings to the broader state or national context. Nevertheless, this strategic sampling approach was deliberate, aimed at capturing diverse perspectives across dissimilar districts to maximise the variability in the implementation of the ISC approach. A second limitation concerns the scope of data collection, which primarily involved soliciting insights from a select group of individuals within a single sector. This approach fails to fully encapsulate the perspectives of a wider array of stakeholders tangentially associated with the programme. Moreover, there exists the potential for social desirability bias, wherein participants may tailor their responses to align with perceived societal norms or congruent with the interviewer’s expertise in nutrition programming. This inherent bias could have potentially skewed responses towards specific narratives or viewpoints, thereby influencing the integrity of the data. Additionally, the interviewer’s familiarity with the subject matter might have inadvertently influenced question framing or interpretation of responses, thereby potentially affecting participants’ viewpoints. To mitigate these concerns, we employed an open-ended questioning approach and emphasised the confidential nature of the study to foster an environment conducive to candid and truthful responses. Furthermore, the interviewer underwent comprehensive training to minimise personal biases and uphold impartiality in both question formulation and data interpretation processes (e.g., reflexivity journal).

We used the National Nutrition Mission as an entry point to study intersectoral collaboration. Our study findings contribute to the growing evidence of intersectoral collaborative approaches, and many of the factors identified within the present study are likely to be found in similar contexts, particularly LMICs or from Asian countries. In its initial stage, we employed a comprehensive approach to examining the factors that promote or hinder ISC at different administrative levels of the health and nutrition system. Our study shifts the focus from traditional structural-centric views to a more nuanced understanding that includes the relational dynamics essential for ISC success. Its significance lies in enhancing the understanding of ISC operational dynamics and informing policy and practice, offering a foundation for future research in diverse geographical contexts and quantitative analysis of ISC outcomes. This research is instrumental in guiding strategies for ISC effectiveness, emphasising both structural aspects and the human element of trust and collaboration in public health initiatives. Furthermore, there is an avenue for further investigation utilising social network analysis to dissect the intricacies of collaboration networks and their impact on operational and outcomes.

## Conclusion

Despite strong calls for intersectoral collaboration globally, operationalising was found to be rather challenging in the field. Based on the identified barriers and facilitators in this study, for a successful ISC, a systematic approach with a clearer articulation of sectoral roles is imperative. ISC efforts should engage stakeholders from different sectors and develop budgeted action plans from federal, provincial and local institutions. Wider stakeholder consultation at the time of formulation and systematic dissemination of ISC plans among the stakeholders of different sectors, along with institutionalising regular reviews and evaluations, would influence fostering and sustaining intersectoral collaborations.

## Data Availability

No datasets were generated or analysed during the current study.
